# Genetic Diversity and Structure of *Lolium* Species Surveyed on Nuclear Simple Sequence Repeat and Cytoplasmic Markers

**DOI:** 10.3389/fpls.2017.00584

**Published:** 2017-04-21

**Authors:** Xuanli Guan, Nana Yuyama, Alan Stewart, Chenglong Ding, Nengxiang Xu, Takako Kiyoshi, Hongwei Cai

**Affiliations:** ^1^Department of Plant Genetics, Breeding and Seed Science, College of Agronomy and Biotechnology, China Agricultural UniversityBeijing, China; ^2^Laboratory of Crop Heterosis and Utilization, Ministry of EducationBeijing, China; ^3^Beijing Key Laboratory of Crop Genetic Improvement and Genome, Ministry of AgricultureBeijing, China; ^4^Forage Crop Research Institute, Japan Grassland Agricultural and Forage Seed AssociationNasushiobara, Japan; ^5^PGG Wrightson SeedsChristchurch, New Zealand; ^6^Institute of Livestock Science, Jiangsu Academy of Agricultural SciencesNanjing, China; ^7^Forage Crop Biotechnology Research Team, National Institute of Livestock and Grassland ScienceNasushiobara, Japan

**Keywords:** *Lolium*, genetic diversity, population structure, nuclear SSR, cytoplasm gene

## Abstract

To assess the genetic diversity and population structure of *Lolium* species, we used 32 nuclear simple sequence repeat (SSR) markers and 7 cytoplasmic gene markers to analyze a total of 357 individuals from 162 accessions of 9 *Lolium* species. This survey revealed a high level of polymorphism, with an average number of alleles per locus of 23.59 and 5.29 and an average PIC-value of 0.83 and 0.54 for nuclear SSR markers and cytoplasmic gene markers, respectively. Analysis of molecular variance (AMOVA) revealed that 16.27 and 16.53% of the total variation was due to differences among species, with the remaining 56.35 and 83.47% due to differences within species and 27.39 and 0% due to differences within individuals in 32 nuclear SSR markers set and 6 chloroplast gene markers set, respectively. The 32 nuclear SSR markers detected three subpopulations among 357 individuals, whereas the 6 chloroplast gene markers revealed three subpopulations among 160 accessions in the STRUCTURE analysis. In the clustering analysis, the three inbred species clustered into a single group, whereas the outbreeding species were clearly divided, especially according to nuclear SSR markers. In addition, almost all *Lolium multiflorum* populations were clustered into group C4, which could be further divided into three subgroups, whereas *Lolium perenne* populations primarily clustered into two groups (C2 and C3), with a few lines that instead grouped with *L. multiflorum* (C4) or *Lolium rigidum* (C6). Together, these results will useful for the use of *Lolium* germplasm for improvement and increase the effectiveness of ryegrass breeding.

## Introduction

The genus *Lolium* comprises nine species representing both outbreeding and inbreeding species (Terrell, 1968; Scholz et al., 2000), of which the most commonly used species are *Lolium perenne* L. (perennial ryegrass) and *Lolium multiflorum* L. (Italian ryegrass or annual ryegrass). These two species produce high yields, are widely adaptable, and have high nutritional value; they are the most important pasture-grass species for cool temperate grassland agriculture, with large areas of cultivation in the British Isles, Denmark, Northern Europe, New Zealand, Southeastern Australia, and other countries (Guthridge, 2001). In addition, *L. perenne* is noteworthy for its use as turf in golf courses and lawns worldwide. Another outbreeding species, *Lolium rigidum*, is typically regarded as a forage species, like *L. multiflorum*. In contrast, the three of inbreeding species—*Lolium temulentum, Lolium persicum*, and *Lolium remotum*—are considered to be weedy species of wheat, oat, and flax fields, respectively.

The species within the genus *Lolium* (ryegrass) are all diploid (2n = 2x = 14), except for some improved tetraploid cultivars of *L. perenne* and *L. multiflorum*. A two-locus self-incompatibility system in the *Lolium* outbreeding species (Cornish et al., 1979) maintains the obligate outbreeding habit. The self-incompatibility and outbreeding features increase genetic variation and complexity in the genus *Lolium*. Most studies have indicated that the genus can be divided into two groups—an inbreeding group and an outbreeding group—according to morphologic features (Bulinska-Radomska and Lester, 1985; Loos, 1993; Bennett et al., 2000), seed proteins (Bulinska-Radomska and Lester, 1985), isozymes and interspecific hybridization (Charmet and Balfourier, 1994; Charmet et al., 1996), and molecular markers such as restriction fragment length polymorphism (RFLP) and random amplified polymorphic DNA (RAPD) markers (Charmet et al., 1997), internal transcribed spacer (ITS) rDNA (Charmet et al., 1997; Gaut et al., 2000; Catalan et al., 2004), sequence-related amplified polymorphism (SRAP) markers (Cheng et al., 2016a), and chloroplast DNA (Balfourier et al., 2000; Cheng et al., 2016b). In contrast, although both *L. rigidum* and *L. perenne* are outbreeding species, among 51 natural populations sampled throughout Europe and the Middle East, most of the *L. rigidum* populations clustered with those of the three inbred species (*L. temulentum, L. persicum*, and *L. remotum*), whereas the *L. perenne* populations could be divided between two different clusters on the basis of chloroplast DNA markers (Balfourier et al., 2000). Cresswell et al. (2001) used amplified fragment length polymorphism (AFLP) markers to analyze three populations of *L. perenne*, four of *L. multiflorum*, and nine of their hybrid, *L*. × *hybridum*, from locations across Portugal and found that the three populations of *L. perenne* formed a discrete cluster that was widely separated from all other populations, whereas, *L*. × *hybridum* populations formed two distinct groups, one of which was similar to and overlapped with *L. multiflorum*, and the second formed a separate cluster.

Due to simple sequence repeats (SSRs) markers exhibit co-dominant inheritance, multiple allelic complexity, it have proven to be highly effective for the evaluation of genetic variation, and it can be detected high polymorphism levels within and between populations. For these reasons, SSRs have continued to be the molecular marker system of choice for studies of plant genetic diversity (Matsuoka et al., 2002; Balfourier et al., 2007; Blair et al., 2009). In the *Festuca–Lolium* complex, SSR markers based genetic diversity studies also have been reported on single or a few species including tall fescue and meadow fescue (Hand et al., 2012), *L. temulentum* (Kirigwi et al., 2008; Hirata et al., 2011), *Lolium persicum* (Sharifi Tehrani et al., 2008; Hirata et al., 2011), and *L. perenne* (Wang et al., 2009), but no reports on all nine species of genus *Lolium*.

*Festuca*, a close related genus to *Lolium*, is one of the largest genera in the grass family (Poaceae; Clayton and Renvoize, 1986; Tzvelev, 1989), with a global distribution in temperate and alpine regions including some economically important species like *Festuca arundinacea* (tall fescue), *Festuca pratensis* (meadow fescue), and *Festuca rubra* (red fescue) used as forage or lawns. Compared with the *Lolium* species, most *Festuca* species are perennial outbreeders, but they display wide variation in ploidy level, ranging from diploid to decaploid. A better understanding of phylogenetic relationships within the species of *Festuca*–*Lolium* complex would not only be very useful for future species conservation and for improved collection knowledge, but would also greatly assist future for age grass breeding programs (Cheng et al., 2016b). A number of phylogenetic analysis of *Festuca–Lolium* complex have been reported based on ITS sequence (Gaut et al., 2000; Catalan et al., 2004), chloroplast gene sequence (Catalan et al., 2004; Cheng et al., 2016b), nuclear genes (Hand et al., 2010) and SRAP markers (Cheng et al., 2016a), and these reports indicated the *Festuca–Lolium* complex can be derived into fine-leaved fescue group and broad-leaved fescue group, and the *Lolium* species were grouped into broad-leaved fescue group.

Most of the previous studies focused on the phylogenetic relationships among species included in the *Festuca*–*Lolium* complex, evaluating a few individuals of each species, rather than on the genetic divergence within the same species. In the current study, to investigate the relationships among nine species of *Lolium* and the genetic diversity within these species, we used nuclear SSR markers and cytoplasmic gene polymerase chain reaction (PCR) markers to characterize a total of 357 individuals from 162 accessions of nine *Lolium* species. Our findings likely will be useful for future genetic diversity studies of *Lolium*.

## Materials and methods

### Plant materials

A total of 357 individuals sampled from 162 accessions (1–3 individuals for each outbreeding species and 1 individual per inbreeding species) and representing nine species of *Lolium* were used. Because the cytoplasmic gene showed matrilineal inheritance, the open pollination progenies of same accession will have same cytoplasmic genotypes, so we used only one individual for each accession for the cytoplasmic gene analysis. Most materials were kindly provided by the United States National Plant Germplasm System, GRIN–USDA, ARS; the remaining samples were from the Forage Crop Research Institute, Japan Grassland Agriculture and Forage Seed Association (Table 1, Table S1). The species classification used was as received.

**Table 1:** **Materials used in this study**.

**Species name**	**No. of accession**	**No. of individuals**
*L. rigidum*	26	76
*L. temulentum*	34	34
*L. persicum*	19	19
*L. remotum*	2	2
*L. perenne*	32	86
*L. multiflorum*	44	131
*L. canariense*	2	4
*L. edwardii*	1	1
*L. subulatum*	2	4
Total	162	357

### Genomic DNA extraction

Total DNA was extracted from fresh leaves by using the cetyl trimethylammonium bromide (CTAB) method (Murray and Thompson, 1980). DNA concentrations were estimated by spectrophotometry (NanoDrop 2000, Thermo Fisher Scientific, Waltham, MA, USA), and the final concentration of each DNA sample was adjusted to 25 ng/μL.

### PCR amplification

In total, 32 nuclear SSR primers from *L. multiflorum* (Table S2, Hirata et al., 2006) distributed in the seven linkage groups of entire genome were used to analyze nuclear diversity. PCR for nuclear SSR markers were performed in a total volume of 10 μL containing 0.5 unit of *Taq* polymerase (Tiangen, Beijing, China), 1.0 μL of 10 × *Taq* DNA polymerase buffer, 0.4 μL of 500 μM dNTPs, 1 pmol M13-taied forward primer, 5 pmol reverse primer, 5 pmol M13 primer, and 20 ng total DNA. The M13 primer (5′ CACGACGTTGTAAAACGAC 3′) was labeled with IRD700 or IRD800 fluorescent dye (Li-COR, Lincoln, NE, USA), and for the M13-taied forward primer, the M13 primer sequence was added to the 5′ end of the forward primer. PCR amplification was performed according to the following program: 95°C for 5 min; then 2 cycles of 95°C for 1 min, 65°C for 1 min, and 72°C for 1.5 min; followed by 10 cycles of 95°C for 1 min, 65–55°C for 1 min decreasing by 1°C per cycle, and 72°C 1.5 min; then 30 cycles of 95°C for 1 min, 55°C 1 min, and 72°C for 1.5 min; followed by 72°C for 7 min; and ending with a 4°C hold. PCR products were confirmed by a LI-COR 4300 DNA Sequencer (LI-COR, Lincoln, NE, USA).

A total of six chloroplast and one mitochondrial primer pairs developed from tabaco, spinach, rice, and carrot (Table S3; Nakamura et al., 1998; Robison and Wolyn, 2002; Kishimoto et al., 2003) that revealed polymorphism were selected for use after the screening of 47 chloroplast and 32 mitochondrial primer pairs (data not shown). For the cytoplasmic gene markers, 160 individuals of 160 accession were used (one individual for each accession). Each PCR was performed in a total volume of 20 μL containing 1.0 unit of *Taq* polymerase (Tiangen, Beijing, China), 2.0 μL of 10 × *Taq* DNA polymerase buffer, 0.4 μL of 500 μM dNTPs, 2 μM of each primer, and 40 ng total DNA. PCR conditions were as follows: 35 cycles of 30 s at 95°C for denaturation, 30 s at 55°C for annealing, and 1 min at 72°C for extension, followed by holding at 4°C. PCR products were separated on 2% agarose gels.

### Phylogenetic and population structure analysis

The number of alleles, the number of genotypes, polymorphism information content (PIC) per locus, pair-wise comparisons of species genetic distance (Nei et al., 1983), and *F*_*st*_ (genetic differentiation) were calculated by using PowerMarker version 3.25 (Liu and Muse, 2005). Phylogenetic trees were constructed by using the unrooted neighbor-joining tree method based on shared genetic distance in PowerMarker and were displayed by using the program MEGA 4.0 (Tamura et al., 2007). Population structure was estimated by using the model-based program STRUCTURE 2.2 (Pritchard et al., 2000; Falush et al., 2003). The analysis had a burn-in length of 10,000 iterations and a run length of 100,000 iterations, and the model allowed for admixture and correlated allele frequencies. At least 20 runs of structure estimation were performed by setting the number of populations (K) from 2 to 10, and the average likelihood value, L(K), across all runs was calculated for each K. The model choice criterion to detect the most probable value of K was ΔK, which is an *ad hoc* quantity related to the second-order change of the log probability of data with regard to the number of clusters inferred by STRUCTURE (Evanno et al., 2005). Analysis of molecular variance (AMOVA) was calculated by using ARLEQUIN ver. 3.5.2 (Excoffier et al., 2005) based on the haplotypic data mode.

## Results

### Genetic diversity

All 32 nuclear SSR primers tested were polymorphic across the 357 individuals, and 755 alleles were detected overall (Table S2). The average number of alleles per locus was 23.59, ranging from 9 (LMSSR09-07G) to 36 (IRGSSR252). The average number of genotypes was 69.13, ranging from 12 (LMSSR09-07G) to 120 (LMSSRST7G4). PIC varied from 0.48 for LMSSR09-07G to 0.95 for LMSSRST12H9, with an average of 0.83. This value is much higher than that obtained by Roldan-Ruiz et al. (2001) by AFLP analysis (PIC, 0.28) and Bolaric et al. (2005) by using RAPD markers (PIC, 0.41).

The seven pairs of cytoplasmic polymorphic primers revealed a total of 37 alleles across the 160 accessions (Table S3). The average number of alleles per locus and the average number of genotypes were both 5.29, ranging from 3 (*atpH*) to 7 (*psaA*). The average PIC-value was 0.54, ranging from 0.14 (ORF100-1) to 0.76 (*petA*). The average number of alleles per locus and PIC determined by using the cytoplasmic gene markers were smaller than those from the nuclear SSR primers.

The genetic diversity for each species is listed in Table 2; four species—*L. remotum, Lolium canariense, Lolium edwardii*, and *Lolium subulatum—*were excluded because only few individuals were evaluated for each. According to the nuclear primers, *L. multiflorum* had the highest PIC (0.83) and *L. persicum* had the lowest PIC (0.21). In contrast, *L. rigidum* had the highest PIC (0.52) and *L. persicum* had the lowest PIC (0.27) on the basis of the six chloroplast gene markers. These results indicate that genetic diversity is higher in the three out-crossing species, *L. multiflorum, L. perenne*, and *L. rigidum*, but is lower in the inbreeding species *L. persicum* and *L. temulentum*.

**Table 2:** **The genetic diversity for five major *Lolium* species**.

**Species**	**Nuclear SSR marker**						**Chloroplast gene marker**					
	**No. of sample**	**AN**	**AF**	**GD**	**H**	**PIC**	**No. of Sample**	**AN**	**AF**	**GD**	**H**	**PIC**
*L. rigidum*	76	17.88	0.30	0.84	0.30	0.82	26	4.50	0.58	0.56	0.00	0.52
*L. temulentum*	34	4.28	0.74	0.36	0.03	0.34	34	3.17	0.74	0.35	0.00	0.32
*L. persicum*	19	2.34	0.83	0.23	0.03	0.21	19	2.17	0.75	0.33	0.00	0.27
*L. perenne*	86	14.72	0.40	0.74	0.23	0.72	32	4.17	0.56	0.55	0.00	0.51
*L. multiflorum*	131	17.34	0.28	0.84	0.30	0.83	42	3.83	0.58	0.50	0.00	0.44

*No. of sample: Number of samples used. AN, Number of alleles; AF, Frequency of major allele; GD, Genetic diversity; H, Heterozygosity; PIC, Polymorphism information content*.

### Genetic relationships among the nine species

According to the nuclear SSR markers, Nei's genetic distance ranged from 0.25 to 0.76 among all pairs of the nine *Lolium* species (Table 3); the greatest genetic distance occurred between *L. edwardii* and *L. persicum*, whereas the smallest was observed between *L. multiflorum* and *L. perenne*. Analysis of molecular variance (AMOVA) revealed that 16.27% of the total variation was due to differences among species, with the remaining 56.35% due to differences within species and 27.39% due to differences within individuals (Table S6). Pair-wise estimates of *F*_*st*_ indicated a high degree of differentiation among the nine species, and the trend was similar to that for genetic distance. As for genetic distance, the highest pair-wise *F*_*st*_-value occurred between *L. edwardii* and *L. persicum* (0.72); the lowest pair-wise *F*_*st*_-value was between *L. multiflorum* and *L. rigidum* (0.06). All pair-wise *F*_*st*_-values for the nine species were significant (*P* < 0.001).

**Table 3:** **Pair-wise estimates of F_st_ (above diagonal) and Nei's genetic distance (below diagonal) based on nuclear SSR markers among nine species**.

**Species**	***L*. *rigidum***	***L*. *temulentum***	***L*. *persicum***	***L*. *remotum***	***L*. *perenne***	***L*. *multifloru***	***L*. *canariense***	***L*. *edwardii***	***L*. *subulatum***
*L. rigidum*		0.26	0.3	0.22	0.07	0.06	0.23	0.16	0.14
*L. temulentum*	0.52		0.36	0.28	0.3	0.26	0.58	0.57	0.49
*L. persicum*	0.57	0.3		0.58	0.35	0.29	0.71	0.72	0.62
*L. remotum*	0.63	0.31	0.48		0.26	0.23	0.7	0.7	0.46
*L. perenne*	0.27	0.52	0.6	0.61		0.09	0.27	0.2	0.16
*L. multiflorum*	0.33	0.56	0.63	0.65	0.25		0.24	0.18	0.16
*L. canariense*	0.58	0.67	0.72	0.74	0.57	0.6		0.58	0.48
*L. edwardii*	0.65	0.7	0.76	0.72	0.62	0.67	0.49		0.4
*L. subulatum*	0.55	0.68	0.74	0.72	0.48	0.59	0.66	0.69	

On the basis of the six chloroplast gene markers, Nei's genetic distance ranged from 0.06 to 0.72 (Table 4), with the greatest distance between *L. edwardii* and *L. subulatum* and the smallest between *L. multiflorum* and *L. perenne*. The overall AMOVA indicated that 16.53% of the variation was due to differences among species; the remaining 83.47% was due to differences within species (Table S7). Pair-wise estimates of *F*_*st*_ showed that the highest degree of differentiation occurred between *L. persicum* and *L. subulatum* (0.68), with the least differentiation between *L. multiflorum* and *L. canariense* (0.02).

**Table 4:** **Pair-wise estimates of F_st_ (above diagonal) and Nei's genetic distance (below diagonal) based on chloroplast gene markers among nine species**.

**Species**	***L*. *rigidum***	***L*. *temulentum***	***L*. *persicum***	***L*. *remotum***	***L*. *perenne***	***L*. *multiflorum***	***L*. *canariense***	***L*. *edwardii***	***L*. *subulatum***
*L. rigidum*		0.20	0.43	0.40	0.15	0.20	0.22	0.47	0.43
*L. temulentum*	0.17		0.23	0.40	0.27	0.23	0.27	0.42	0.33
*L. persicum*	0.34	0.15		0.53	0.31	0.30	0.34	0.45	0.68
*L. remotum*	0.45	0.34	0.16		0.24	0.18	0.25	0.14	0.48
*L. perenne*	0.24	0.24	0.31	0.46		0.13	0.15	0.27	0.24
*L. multiflorum*	0.19	0.18	0.30	0.40	0.06		0.02	0.15	0.17
*L. canariense*	0.55	0.60	0.50	0.56	0.30	0.40		0.23	0.21
*L. edwardii*	0.41	0.38	0.32	0.22	0.37	0.35	0.65		0.38
*L. subulatum*	0.52	0.55	0.51	0.63	0.37	0.51	0.47	0.72	

### Analysis of population structure

**Figure 1:**
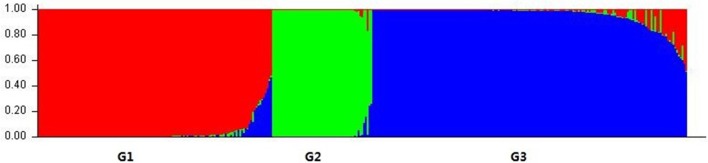
**Population structure of *Lolium* species based on 32 nuclear SSR markers (*K* = 3)**. Bar plot was sorted by Q-values in single line.

We used a model-based clustering method for multi-loci genotype data to infer the population structure and assign individuals to populations by using STRUCTURE. According to L (K) and ΔK, the model-based simulation of population structure using nuclear SSR markers showed that the optimal number of populations was 3, meaning that the ancestry of each individual was inferred from the Q-value and classified into one of three groups, as inferred from the model, here denoted as G1, G2, and G3, respectively (Figure 1; Figure S1; Table S4). Group G1 consisted of 129 individuals, including most of those of *L. multiflorum* and a few of *L. perenne*; G2 comprised the 55 individuals from all three inbreeding species, and G3 contained the 173 remaining individuals, the majority of which belonged to *L. rigidum* and *L. perenne*; the three rare species of *L. subulatum* (4 individuals), *L. canariense* (4 individuals), and *L. edwardii* (1 individual) were also grouped into G3.

**Figure 2:**
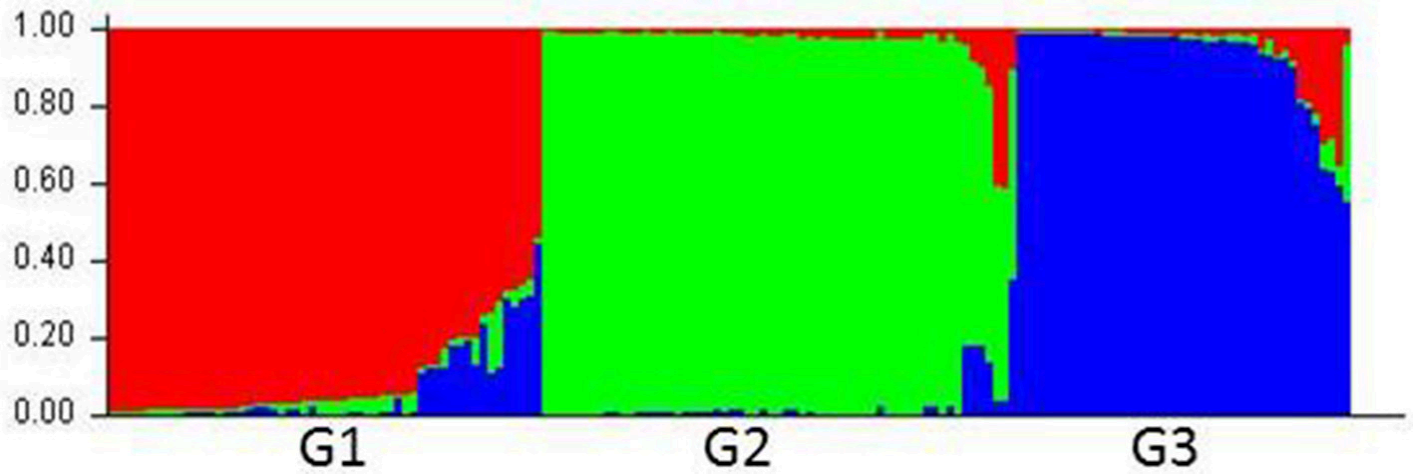
**Population structure of *Lolium* species based on six chloroplast gene markers (*K* = 3)**. Bar plot was sorted by Q-values in single line.

Compared with the results from the nuclear SSR markers, the population structure inferred by using cpDNA markers was not delineated as clearly. The 160 accessions were grouped optimally into three subpopulations (*K* = 3), denoted as G1, G2, and G3, respectively (Figure 2; Figure S2; Table S5).

**Figure 3:**
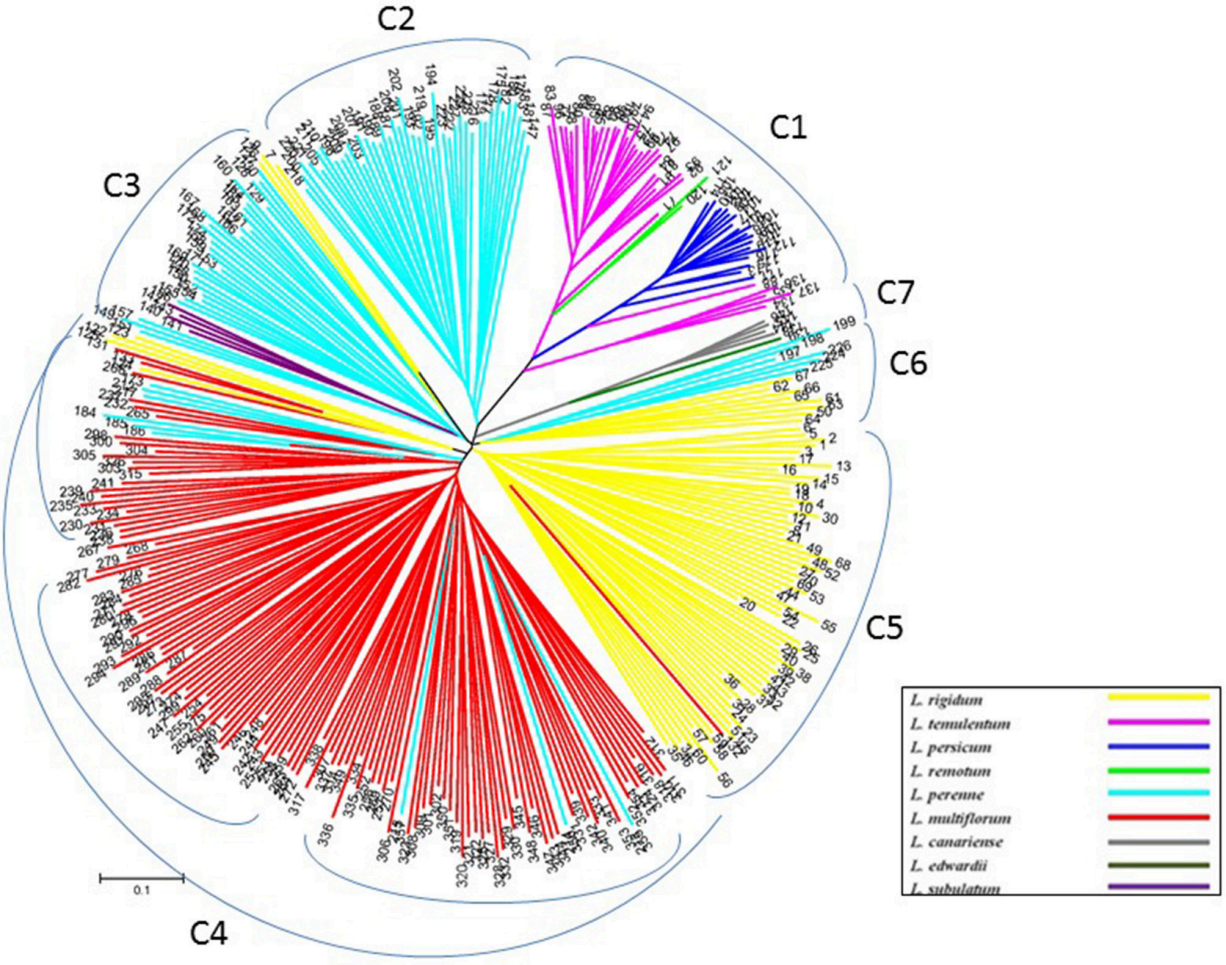
**Unrooted neighbor-joining tree comprising 357 individuals from 162 accessions of *Lolium*, according to 32 nuclear SSR markers**.

**Figure 4:**
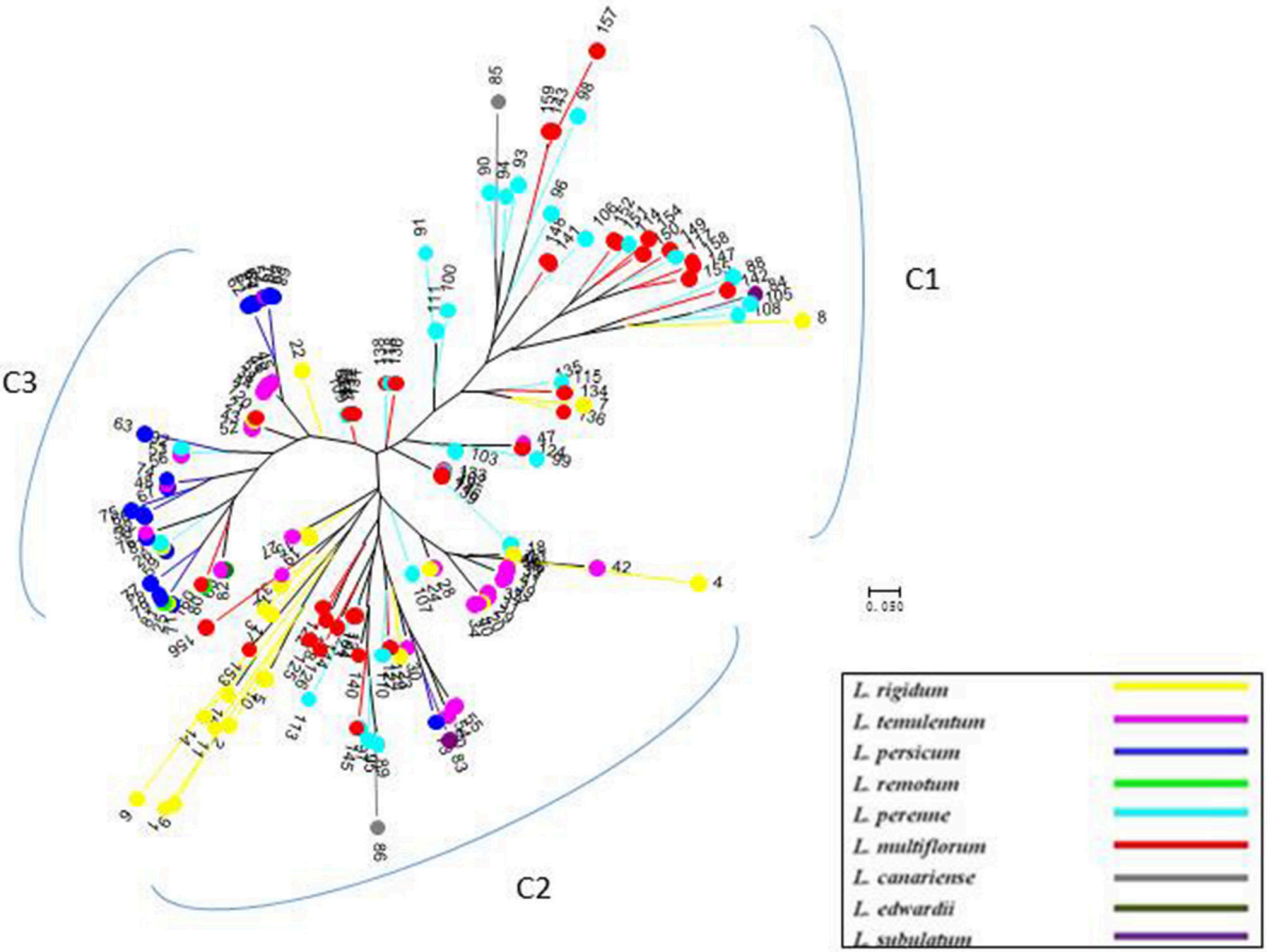
**Unrooted neighbor-joining tree of 160 accessions of *Lolium*, according to six chloroplast gene markers**.

To verify the results of the STRUCTURE analysis, unrooted neighbor-joining trees of 357 individuals and 160 accessions were constructed based on Nei's genetic distance by using nuclear SSR markers and cpDNA gene markers, respectively (Figures 3, 4, and Table S1). With few exceptions, the neighbor-joining tree showed that the *Lolium* materials could be differentiated according to their subspecies affiliation. In addition, compared with the results from the STRUCTURE analysis, the materials were better differentiated and more clearly visualized in the neighbor-joining tree. For example, three inbreeding species were clustered into one group (C1), whereas another inbreeding species, *L. subulatum*, was grouped with *L. perenne* (C3). Almost all *L. multiflorum* individuals were allocated into group C4, which seemed to consist of three subgroups. Populations of *L. perenne* clearly clustered into two groups (C2 and C3), although a few lines of *L. perenne* were grouped with *L. multiflorum* (C4) and *L. rigidum* (C6). Interestingly, two minor species, *L. canariense* (represented by four individuals) and *L. edwardii* (one individual), both from Spain, were grouped as C7 (Figure 3).

Compared with nuclear SSR markers, the clustering analysis based on cpDNA gene markers detected three groups, but the subspecies affiliation were not so clear (Figure 4), although most of the inbreeding species, particularly *L. persicum* populations, were included in group C3, and most *L. perenne* and *some L. multiflorum* were grouped as C1.

## Discussion

SSR marker were easily transferred between *Festuca–Lolium* complex (Rouf Mian et al., 2005; Hirata et al., 2006, 2011; Kirigwi et al., 2008; Sharifi Tehrani et al., 2008; Hand et al., 2012), in this study, SSR markers form Italian ryegrass were used, in our results, the 32 nuclear SSR markers were successfully amplified in all nine *Lolium* species used, and showed higher genetic diversity and PIC-values in out-crossing species than inbreeding species, suggesting the usefulness of these SSR markers in *Lolium* genetic diversity study. On the other hands, the organellar genome is usually well conserved among different plant species, and organellar markers are used as tools for studies of population genetics and evolution and for phylogenic analysis (Dumolin-Lapegue et al., 1997). We have tested a total of 38 primer pairs from chloroplast genes and 30 primer pairs from mitochondrial genes designed from rice and other species, and almost all the primers could provide single clear amplification products in Italian ryegrass (Cai et al., 2009). In this study, seven cytoplasmic markers were from tabaco, spinach, rice, and carrot, and these markers showed a moderate polymorphism in nine *Lolium* species, although other tested cytoplasmic primers don't detected polymorphism by using agarose electrophorese (data not shown), to get more polymorphism using these cytoplasmic primers, it may need to detect polymorphism by digest the amplification products using four-base cutter restriction endonuclease like *Alu*I, *Hae*III, *Rsa*I, *Hha*I, *Msp*I, and *Mse*I, or by finding of SNP by sequencing of the PCR products.

Although many reports address the phylogenetic relationships of the *Festuca–Lolium* complex (Charmet et al., 1997; Gaut et al., 2000; Hand et al., 2010; Cheng et al., 2016a,b), few are molecular studies of larger numbers of *Lolium* samples. In contrast, the current study evaluated a total of 357 individuals from 160 accessions representing both the five major species as well as four minor species—*L. remotum, L. canariense, L. edwardii*, and *L. subulatum*—to give an overall view of the relationships among the *Lolium* species at the molecular level. Our results were mostly in agreement with those from a morphological study (Loos, 1993) and an isozyme study (Charmet et al., 1996). Loos (1993) indicated that the two inbreeding species *L. loliaceum* (*L. subulatum*) and *L. remotum* do not form a distinct group and that *L. remotum* is intermediate between the cross-breeding species and the other inbreeding species, whereas *L. loliaceum* is in a somewhat isolated position, more closely related to *L. rigidum*. However, in our results, the *L. remotum* populations grouped with the inbreeding species and close to *L. temulentum*, whereas *L. subulatum* was not grouped with *L. rigidum* but rather close to *L. perenne*. In contrast, Balfourier et al. (2000) reported that *L. perenne* populations can be divided into two different clusters on the basis of chloroplast DNA markers. Similarly, using nuclear SSR markers, we also detected two groups of *L. perenne*: group C3 materials were mainly from the Middle East and Northern Europe, whereas those in group C2 were from other regions of Europe primarily; in addition, a few *L. perenne* accessions were grouped with *L. multiflorum* (C4) and *L. rigidum* (C6). Moreover, *L. multiflorum* (C4) seemed to be divided into three subgroups, which have not been reported previously.

Due to adapting to differences in the ecological environment from place to place, *Lolium* seems to have formed different geographic ecological groups during long-term migration and evolution (Terrell, 1968). In our study, although the two *L. perenne* groups show some geographic evolution tendency according to the nuclear SSR-based clustering, the relationships between the population structures with geographic origin were rather indistinct, especially for the cytoplasmic gene markers.

The contrasted patterns of inheritance of organelle (cpDNA and mtDNA) and nuclear genes can be used to unravel the complexity of gene flow in plants, as they are predicted to result in very different distribution of genetic diversity within and among populations (Birky et al., 1989; Petit et al., 1993). Petit et al. (2005) compared the genetic structure based on chloroplast DNA (cpDNA), mitochondrial DNA (mtDNA) and nuclear markers from a data set of 183 species belonging to 103 genera and 52 families, they concluded that maternally inherited genomes (cpDNA and mtDNA) experience considerably more subdivision than paternally or biparentally inherited genomes (nuclear genes). *G*_ST_ at cpDNA and mtDNA markers covary narrowly when both genomes are maternally inherited, whereas *G*_ST_ at paternally and biparentally inherited markers also covary positively but more loosely and *G*_ST_ at maternally inherited markers are largely independent of values based on nuclear markers. In this study, we used both nuclear SSR markers and cpDNA gene markers for estimation of the population differentiation, but the *Fst*-values of nuclear SSR markers and cpDNA gene markers-based were not clearly different. The genetic diversity and PIC-values for different marker types were also no clear difference detected in same species (Table 2). For the AMOVA results of two types of markers were comparable, nuclear SSR markers revealed that 16.27% of the total variation was due to differences among species, with the remaining 56.35% due to differences within species and 27.39% due to differences within individuals, but the cpDNA gene markers detected 16.53% of the variation was due to differences among species; the remaining 83.47% was due to differences within species, and no variation were from the differences within individuals, because no heterozygosity detected from cpDNA gene markers. Wang et al. (2009) has reported that genetic variation among (8.7%) and within populations (91.3%) in eight forage perennial ryegrass populations comprising 48 individual plants per population genotyped with 29 SSR marker loci. Compared with this result, a larger value of among species and smaller value of within species in our study were detected, because we used both inbreeding and out-crossing species of *Lolium*, which has larger genetic difference.

In this study, we combined the nuclear SSR markers and cytoplasmic gene markers to analyze the genetic diversity and structure among nine *Lolium* species. We found clear differentiation between *L. perenne* populations as well as several tendencies toward differentiation within *L. multiflorum*. These results will be useful for future species conservation and for improved collection knowledge, and will be also useful for efficiently use of *Lolium* germplasm for ryegrass breeding.

## Author contributions

HC: designed the experiments; XG, NY, CD, and NX: performed the experiments; AS and TK: collected materials; XG and HC: analyzed the data; and GX and CH: wrote the manuscript.

### Conflict of interest statement

The authors declare that the research was conducted in the absence of any commercial or financial relationships that could be construed as a potential conflict of interest.

## Supplementary material

Figure S1 **Population structure of *Lolium* species based on 32 nuclear SSR markers (*K* = 3)**. Bar plot was sorted by Q-values in multiple lines.

Figure S2 **Population structure of *Lolium* species based on six chloroplast gene markers (*K* = 3)**. Bar plot was sorted by Q-values in multiple lines.

Table S1 **The materials used in this study**.

Table S2 **Nuclear SSR markers information used in this study**.

Table S3 **Cytoplasm gene markers information used in this study**.

Table S4 **Summary statistics for each group (*K* = 3) in Figure 1**.

Table S5 **Summary statistics for each group (*K* = 3) in Figure 2**.

Table S6 **Results of AMOVA using 32 nuclear SSR markers and 357 Lolium individuals**.

Table S7 **Results of AMOVA using 6 cpDNA gene markers and 160 Lolium individuals**.
