# Supplementary figures and images for: Genetic Diversity and Structure of Lolium Species Surveyed on Nuclear Simple Sequence Repeat and Cytoplasmic Markers
Source: Front Plant Sci. 2017 Apr 21;8:584. doi: 10.3389/fpls.2017.00584 (PMC5399756; doi:10.3389/fpls.2017.00584)

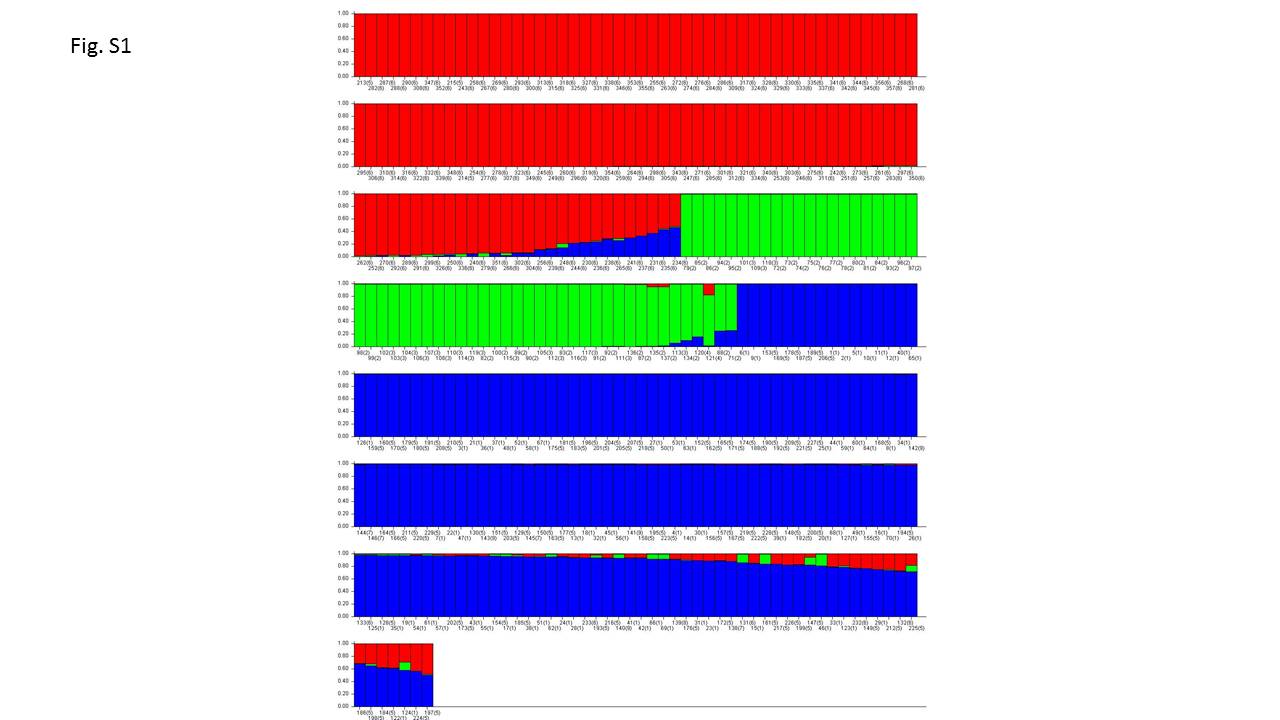

Supplement: Figure S1 — Population structure of Lolium species based on 32 nuclear SSR markers (K = 3). Bar plot was sorted by Q-values in multiple lines. [file Image1.JPEG]

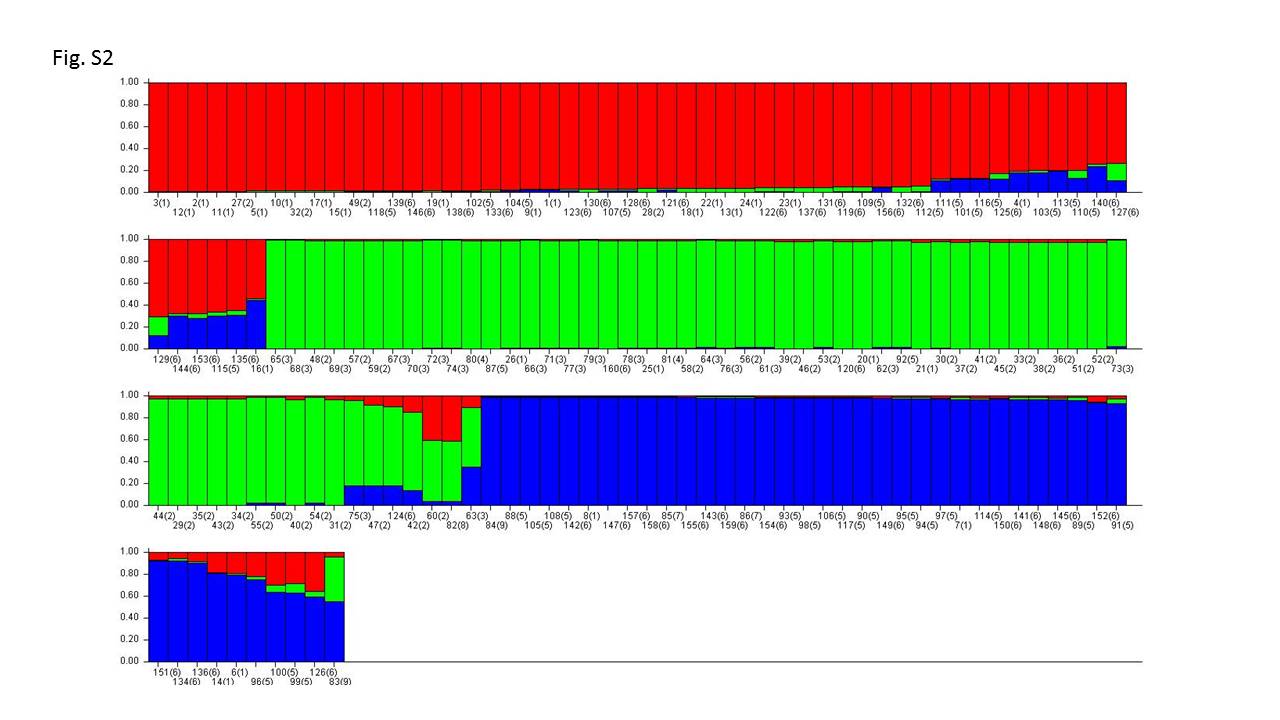

Supplement: Figure S2 — Population structure of Lolium species based on six chloroplast gene markers (K = 3). Bar plot was sorted by Q-values in multiple lines. [file Image2.JPEG]
